# Relationship between Oral Hypofunction, and Protein Intake: A Cross-Sectional Study in Local Community-Dwelling Adults

**DOI:** 10.3390/nu13124377

**Published:** 2021-12-07

**Authors:** Keitaro Nishi, Hiroaki Kanouchi, Akihiko Tanaka, Maya Nakamura, Tomofumi Hamada, Yumiko Mishima, Yuichi Goto, Kenichi Kume, Mahiro Beppu, Hiroshi Hijioka, Hiroaki Tabata, Kazuki Mori, Yoshinori Uchino, Kouta Yamashiro, Yoshiaki Matsumura, Yutaro Higashi, Hyuma Makizako, Takuro Kubozono, Toshihiro Takenaka, Mitsuru Ohishi, Tsuyoshi Sugiura

**Affiliations:** 1 Department of Maxillofacial Diagnostic and Surgical Science, Field of Oral and Maxillofacial Rehabilitation, Graduate School of Medical and Dental Sciences, Kagoshima University, Kagoshima 890-8544, Japan; k9296982@kadai.jp (K.N.); althair@dent.kagoshima-u.ac.jp (A.T.); k6996689@kadai.jp (M.N.); thamada@sagara.or.jp (T.H.); k5782265@kadai.jp (Y.M.); ygoto@dent.kagoshima-u.ac.jp (Y.G.); kkume@dent.kagoshima-u.ac.jp (K.K.); mbeppu@dent.kagoshima-u.ac.jp (M.B.); zio@dent.kagoshima-u.ac.jp (H.H.); k5258228@kadai.jp (H.T.); k4639792@kadai.jp (K.M.); k2309975@kadai.jp (Y.U.); k5124979@kadai.jp (K.Y.); k6917253@kadai.jp (Y.M.); k0970037@kadai.jp (Y.H.); 2 Department of Clinical Nutrition, Graduate School of Comprehensive Rehabilitation, Osaka Prefecture University, Osaka 583-8555, Japan; kano@rehab.osakafu-u.ac.jp; 3 Department of Oral & Maxillofacial Surgery, Hakuaikai Social Medical Corporation, Sagara Hospital, Kagoshima 892-0833, Japan; 4 Department of Physical Therapy, School of Health Sciences, Faculty of Medicine, Kagoshima University, Kagoshima 890-8544, Japan; makizako@health.nop.kagoshima-u.ac.jp; 5 Department of Cardiovascular Medicine and Hypertension, Graduate School of Medical and Dental Sciences, Kagoshima University, Kagoshima 890-8544, Japan; kubozono@m.kufm.kagoshima-u.ac.jp (T.K.); ohishi@m2.kufm.kagoshima-u.ac.jp (M.O.); 6 Tarumizu Municipal Medical Center, Tarumizu Chuo Hospital, Kagoshima 891-2124, Japan; takenaka@tarumizumh.jp

**Keywords:** oral hypofunction, protein intake, sarcopenia, frailty

## Abstract

Few studies have investigated the relationship between nutritional status and comprehensive assessment of oral hypofunction, especially protein intake-related sarcopenia. Thus, we explored these relationships in a large-scale cross-sectional cohort study using the seven-item evaluation for oral hypofunction and Diet History Questionnaire for nutritional assessment. We used the data from 1004 individuals who participated in the 2019 health survey of the residents of Tarumizu City, Kagoshima Prefecture, Japan for analysis. We found that individuals with oral hypofunction were significantly older with a lower skeletal muscle index. Although there were few foods that had a significant difference between the groups with and without oral hypofunction, the consumption of beans and meats was significantly lower in women and men in the oral hypofunction group, respectively. According to the lower limit of the tentative dietary goal defined in Japan, comprehensive evaluation of oral hypofunction was significantly and independently associated with protein intake in both men and women (odds ratio, 1.70; 95% confidence interval, 1.21–2.35). In conclusion, we found that oral hypofunction was associated with targeted protein intake for sarcopenia and frailty prevention in middle-aged and older community-dwelling adults. Comprehensive evaluation of oral function with intervention in cases of hypofunction could inform clinicians to better prevent sarcopenia.

## 1. Introduction

In Japan, the proportion of the population consisting of aged individuals has increased at a rapid pace over the past 30 years, and this proportion is expected to continue to rise, reaching approximately 40% by 2060 [1]. Furthermore, it is anticipated that a 100-year life span will be attainable. However, since there is roughly a 10-year gap between healthy life expectancy and average life expectancy, there is an urgent need to extend healthy life expectancy while simultaneously improving the health of society [2].

Preventing frailty associated with sarcopenia is necessary to increase the number of years lived with high quality; Iijima et al. reported on the importance of disseminating frailty prevention measures and encouraging prevention activities, such as nutritional status and physical activity in a super-aged society [2]. Moreover, the prevention of frailty and sarcopenia reduce medical and healthcare costs [3]. Frailty and sarcopenia have been shown to be related to protein intake; moreover, low protein intake over time is associated with greater muscle mass loss [4,5]. Based on results of cross-sectional studies, it has been reported that adequate energy intake, especially protein, are necessary for the prevention of frailty [6,7,8]. In systematic reviews of longitudinal studies, decreased protein intake was also cited as a risk factor for frailty development and progression [9,10].

In 2020, the Ministry of Health, Labor and Welfare in Japan revised the “Dietary Reference Intakes for the Japanese”. Among the revised content, the lower limit of the “Tentative Dietary Goal for Preventing Lifestyle-related Diseases (DG)” was increased to aid in preventing frailty [11]. This also indicates that the Japanese government is taking steps to support the extension of healthy life expectancy through diet [11,12].

In 2016, the concept of oral hypofunction was proposed by the Japanese Society of Gerodontology [13]. This is a new area of healthcare that consists of seven comprehensive assessment areas to diagnose “oral hypofunction”, which was defined as the condition of poor overall oral function in older adults, and subsequent management to improve oral function. Specifically, the seven evaluation items are poor oral hygiene, oral dryness, reduced occlusal force, decreased tongue-lip motor function, decreased tongue pressure, decreased masticatory function, and deterioration of swallowing function. The evaluation and management of oral hypofunction in older adults has been covered by the Japanese insurance system since 2018, and evidence of poor oral function is gradually accumulating. Studies have reported on the association between oral hygiene and frailty [14], the association between masticatory function and frailty and/or sarcopenia [15,16], the association between tongue pressure and frailty or sarcopenia [17,18], and the association between the number of remaining teeth and frailty [19]; however, the association between oral hypofunction and frailty or sarcopenia has only recently been reported [20,21]. Based on current trends, our research group conducted a study on the methods to examine oral hypofunction proposed by the Japanese Society of Gerodontology, and reported that oral hypofunction is related to frailty, sarcopenia, and mild cognitive impairment [22].

Additionally, there have recently been reports on the association between individual oral status and nutritional status [23,24,25,26,27,28], and the relationship between oral hypofunction and nutritional status using a simple rating scale (Mini Nutritional Assessment^®^-Short Form [MNA-SF]) [29]. However, there are few reports on the association between oral hypofunction and protein intake, which is also related to the development of frailty and sarcopenia.

It is worthwhile to investigate the association between oral hypofunction as a comprehensive evaluation index of oral function and protein intake, which is associated with frailty and sarcopenia. Therefore, we conducted a cross-sectional study of the association between oral hypofunction and dietary and nutritional intake using data from a prospective cohort study of community-dwelling adults in Tarumizu City, Kagoshima Prefecture, Japan, which was piloted in 2017.

## 2. Materials and Methods

### 2.1. Participants

People aged 40 years and older living in Tarumizu city in 2019 were included in this study. We obtained approval from the Clinical Research Ethics Committee of Kagoshima University Hospital (ref no. 170351) prior to beginning the study. Participants provided written consent prior to participation.

**Figure 1:**
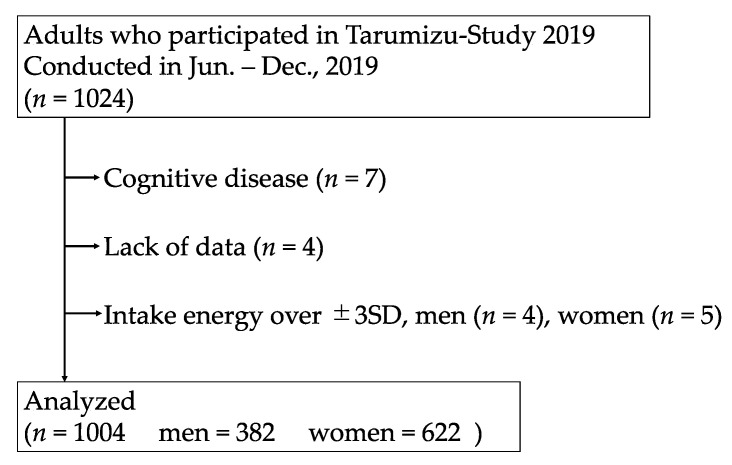
Flowchart for inclusion of study participants.

Criteria for inclusion in the analysis are shown in Figure 1. Those with a history of dementia, lack of data, and >±3 standard deviations (SDs) from the energy intake calculated using the Brief Type Self-administered Diet History Questionnaire (BDHQ) described below were excluded. Ultimately, 1004 participants were included in the analysis.

### 2.2. Diagnosis of Oral Hypofunction

The evaluation and diagnosis of oral hypofunction were based on the 2016 diagnostic criteria of the Japanese Society of Gerodontology with some modifications [13]. The seven-item evaluation was performed and investigated as follows. Table 1 shows the criteria for seven-item evaluation. A diagnosis of oral hypofunction was made when three or more items were assessed in the poor range of function.

Moreover, in this study, in order to determine the degree of oral hypofunction, the total number of items with measured values assessed as functioning poorly, that is, the number of indicators of poor levels of functioning in the oral hypofunction evaluation, was calculated.

#### 2.2.1. Poor Oral Hygiene

Oral hygiene status was assessed based on the Tongue Coating Index (TCI) [30], which was modified slightly in this study as follows. The tongue was divided into the anterior, central, and posterior sections. The degree of tongue coating was evaluated on a two-point scale and recorded as “with tongue coating“ or “without tongue coating” by visual examination. Poor oral hygiene was diagnosed as shown in Table 1.

#### 2.2.2. Oral Dryness

Oral dryness status was measured using a moisture checker (Mucus, Life Co. Ltd., Saitama, Japan). Measurements were taken three times, and the median value was recorded. When as shown in Table 1, the diagnosis of oral dryness was made [13].

#### 2.2.3. Reduced Occlusal Force

According to the diagnostic criteria for oral hypofunction, occlusal force can be assessed using either pressure indicator film measurements or by noting the number of remaining teeth. In this study, the number of remaining teeth was used as an alternative method due to its simplicity and cost-effectiveness. When as shown in Table 1, the diagnosis of decreased occlusal force was made. (Tooth stumps and teeth with mobility 3 were excluded from the count.)

#### 2.2.4. Decreased Tongue-Lip Motor Function

Tongue-lip motor function was measured using an automatic motor function measurement device (Kenkokun Handy; Takei Scientific Instruments, Niigata, Japan). The participants were asked to repeat the syllables /pa/, /ta/, and /ka/ as quickly as possible for 5 s. When as shown in Table 1, a diagnosis of decreased tongue-lip motor function was made.

#### 2.2.5. Tongue Pressure Measurement

Tongue pressure was measured using a tongue pressure-measuring device (JMS tongue pressure measuring instrument, TPM-01; JMS Co. Ltd., Hiroshima, Japan). Measurements were taken three times, and the maximum value was recorded. A diagnosis of decreased tongue pressure was made if the measured value was as shown in Table 1.

#### 2.2.6. Decreased Masticatory Function

Masticatory function was subjectively assessed using a questionnaire based on previous research reports [31]. In the questionnaire, respondents answered an item concerning whether they are able to chew two Japanese hard foods. The participants were diagnosed as having decreased masticatory function if they answered as shown in Table 1.

#### 2.2.7. Assessing of Swallowing Function

The 10-item Eating Assessment Tool (EAT-10) was used to assess swallowing function. A diagnosis of decreased swallowing function was made when as shown in Table 1.

### 2.3. Nutritional Assessment

#### 2.3.1. Brief-Type Self-Administered Diet History Questionnaire (BDHQ)

Nutritional status was assessed using the BDHQ. The BDHQ surveys diets over a one-month period and has been used and validated in other studies [8,32,33]. To minimize the effect of errors due to self-reported nutrient and food intake, and of differences in energy intake between sexes, the residual method was used to normalize the energy levels recorded [33].

#### 2.3.2. Diagnosis of Low Protein Intake

The “Dietary Reference Intake for Japanese” established by the Ministry of Health, Labor and Welfare in Japan was used for diagnosis of low protein intake. The Minister of Health, Labor and Welfare sets standards for energy and nutrients that are desirable to consume in order to maintain and improve the health of the people [11]. Within that standard, the tentative dietary goal (DG) for preventing life-style related diseases was adopted. This is defined as the amount (or range) of nutrients that Japanese people should aim to consume for the primary prevention of lifestyle-related diseases. For protein, the DG is also set; it is determined by the age group, and has a range and upper and lower limits. It is set as a percentage of daily energy intake (% energy). The DG range for protein intake is 13–20% of daily energy intake for those aged 30–49 years, 14–20% for those aged 50–64 years, 15–20% for those aged 65–74 years, and 15–20% for those aged 75 years and older. In this study, participants who did not reach the lower limit of the DG range were diagnosed as having low protein intake. For simplicity, in this study, not reaching the lower limit of the DG range was denoted as “under DG”, while reaching the lower limit was denoted as “over DG”.

### 2.4. Other Surveys

Through a self-administered questionnaire, the participants’ sex, age, current and past illnesses, living circumstances, social circumstances, and educational background were investigated. Physical items included height measurement and weight measurement. Skeletal muscle mass index (SMI) and body mass index (BMI) were also measured using a bioelectrical impedance analysis (BIA) instrument (InBody 470; InBody Japan, Tokyo, Japan; https://www.inbody.co.jp/inbody-470/ accessed on 2 December 2021).

### 2.5. Statistical Analysis

Considering the distribution, the *t*-test and Welch’s test were used for comparison between groups. The Chi-square test was also used for statistical examination between items that were dichotomized. Multivariate analysis using binary logistic regression was performed to examine the effect of oral function decline and oral hypofunction on whether the lower limit of the DG range was reached. Covariates included age, sex, energy intake, BMI, medical history, smoking history, educational background, living circumstances, and social circumstances. All analyses were performed using JMP14.2 (SAS Institute Inc., Cary, NC, USA). Statistical significance was set at *p* < 0.05.

## 3. Results

The analysis included 382 men (mean age, 67.5 ± 11.3 years) and 622 women (mean age, 68.8 ± 10.8 years). In total, 180 (47%) men and 289 (46%) women were diagnosed with oral hypofunction.

### 3.1. Physical Characteristics and Examination Values by Presence or Absence of Oral Hypofunction

Table 2 shows the mean values and standard deviations of each physical characteristic, results of examination for oral hypofunction, and social background in groups with and without oral hypofunction by sex. In both men and women, the oral hypofunction group was significantly older than the no oral hypofunction group with a significantly lower SMI; however, there was no significant difference in BMI between groups. In all examination areas for oral hypofunction, the oral hypofunction group had significantly poorer functioning than the no oral hypofunction group for both men and women. Duration of education (educational background) was significantly shorter in the oral hypofunction group. The number of participants living alone was significantly larger in the oral hypofunction group for both men and women (men *p* = 0.0305, women *p* < 0.0001).

### 3.2. Nutritional Status and Food Intake Based on the Presence or Absence of Oral Hypofunction

The nutritional status and food intake of the participants divided by the presence or absence of oral hypofunction are shown in Table 3. Daily energy intake was significantly higher in the oral hypofunction group in women (*p* = 0.0143). There was no significant difference in protein intake as a percentage of total energy and in g/day between groups with and without oral hypofunction in men and women. Regarding food categories with major protein components, the intake of beans was significantly lower in the oral hypofunction group in women and the intake of meats was significantly lower in the oral hypofunction group in men, while the intake of seafood was higher in the oral hypofunction group in both men and women, although there was no significant difference. Albumin as a serological nutritional assessment index was significantly lower in the oral hypofunction group in both men and women (*p* < 0.0001).

### 3.3. Physical Characteristics, Nutritional Status, and Social Background Based on Protein Intake

The results of physical characteristics, nutritional status, and social background of the participants according to whether protein intake is over or under DG are shown in Table 4. In total, 142 (37%) men and 127 (20%) women had low protein intake (under DG). There were no statistically significant differences in age, height, BMI, or SMI according to sex or DG classification. Daily energy intake was significantly lower in the under-DG group than in the over-DG group in men (men, *p* = 0.0286; women, *p* = 0.9148). For the intake ratio of the three macronutrients to energy intake, protein and fat (% energy) were significantly lower in the under-DG group than in the over-DG group, and carbohydrate intake (% energy) was significantly higher in the under-DG group than in the over-DG group (men and women, *p* < 0.0001). Total protein intake (g/day) was significantly lower in the under-DG group than in the over-DG group in both men and women (*p* < 0.0001), while albumin was not significantly different (men, *p* = 0.5114; women, *p* = 0.6465). Duration of education (educational background) was significantly shorter in the under-DG group than in the over-DG group for both men and women (men, *p* = 0.01; women, *p* = 0.0471).

### 3.4. Oral Hypofunction Evaluation According to Under-DG or Over-DG Protein Intake

Oral hypofunction evaluation results according to under-DG or over-DG protein intake are shown in Table 5. Since there is no sex-specific criteria for oral hypofunction evaluation, the total measurement values for men and women are also shown. There were statistically significant differences in protein intake between the under-DG group and the over-DG group for the number of participants diagnosed with oral hypofunction in the total participants, in men alone, and in women alone by the Chi-square test (men, *p* = 0.0006; women, *p* = 0.0284; total, *p* < 0.0001). The ratio of the number of participants in the under-DG group was larger than that in the over-DG group. The number of items indicating poor functioning scored in the oral hypofunction evaluation was significantly higher in the under-DG group in the total participants, in men alone, and in women alone (men, *p* = 0.0007; women, *p* = 0.0054; total, *p* < 0.0001). Regarding overall oral function evaluation results, values were indicative of poorer functioning than normal in the under-DG group in total, in men alone, and in women alone. There were significant differences in the number of remaining teeth and oral diadochokinesis (ODK) /pa/ rate in women alone (occlusal force, *p* = 0.0054; ODK /pa/, *p* = 0.0041), while there were no significant differences in men alone. In addition, there were statistically significant differences between the number of remaining teeth and ODK /pa/, ODK /ta/, and ODK /ka/ rates in the total participants.

### 3.5. Association with Oral Hypofunction and Protein Intake in Multivariate Binary Logistic Analysis

Table 6 shows the results of the binomial logistic analysis using the availability of protein target intake as the dependent variable and oral function evaluation as the independent variable. In the analysis of men only and of women only, the analysis did not consider covariates because of the relationship between the appropriate number of independent variables and number of samples in the logistic analysis. Since there is no sex-specific criteria for oral function evaluation, the values of the total participants would be more important. Protein intake based on whether it was under or over DG was significantly and independently associated with oral hypofunction in the total participants (odds ratio, 1.70; 95% confidence interval, 1.21–2.35) after adjusting for covariates (age, sex, BMI, energy intake, heart disease, smoking, appetite, living alone, vitality, and education history). Similarly, protein intake based on whether it was under or over DG was significantly and independently associated with the number of poor function indicators in oral hypofunction evaluation in the total participants (odds ratio, 1.24; 95% confidence interval, 1.10–1.40) after adjusting for covariates. Regarding each oral function evaluation, protein intake based on whether it was under or over DG was independently associated with the number of remaining teeth (occlusal force) after adjusting for covariates; however, the odds ratio was not large.

## 4. Discussion

In this study, we investigated the relationship between the nutritional status of participants and presence of oral hypofunction based on the nutritional intake data surveyed using the BDHQ, with a particular focus on protein intake. The oral functional status of participants with under-DG intake was also shown. Although participants diagnosed with oral hypofunction did not have significantly lower protein intake than those without oral hypofunction as shown in Table 3, participants with under-DG protein intake had a significantly higher rate of diagnosis of oral hypofunction and higher number of items indicating poor function in oral hypofunction evaluation than those with over-DG protein intake, as shown in Table 5. Furthermore, protein intake under or over DG was independently associated with oral hypofunction and a number of poor function indicators in oral hypofunction evaluation by multivariate analysis, as shown in Table 6. These results indicate that not all people diagnosed with oral hypofunction have low protein intake. However, most people with oral hypofunction do not reach the high-end target amount (DG) for protein intake from the viewpoint of adequate protein intake to prevent sarcopenia and frailty. Therefore, the diagnosis of oral hypofunction may indicate that it is possible to extract not a few people with low protein intake. Low protein intake has been reported to lead to muscle mass loss in older people [4,5]. It has also been reported to be a factor associated with frailty and sarcopenia; moreover, adequate energy intake, especially protein, has been reported to be important to prevent frailty [6,9,34].

The relationship between tooth contact or number of teeth and nutritional status [35,36], the relationship between number of teeth and masticatory ability [24], and the relationship between masticatory ability and nutritional status [23,24,25,26] have been reported. Based on the results of these studies, masticatory ability may be important to prevent malnutrition, which is one of the gateways to frailty and sarcopenia. However, it has been reported that restoring oral function merely by dental prosthodontic treatment cannot improve general nutritional status [37,38]. Dental clinics are considered to be suitable places to provide nutritional counseling as well as restoring and maintaining oral function [38]; moreover, dietary counseling in addition to dental treatment may be effective in improving nutritional status. This study included participants over 40 years of age and found decreased oral function and protein in this group. Therefore, comprehensive health guidance including nutrition and oral health education for sarcopenia and frailty prevention is necessary beginning from middle age, as Nomura et al. reported [39]. The results of the present study may add further evidence to existing reports that oral hypofunction is associated with low protein intake in the general population living in the community.

In the present study, oral hypofunction is diagnosed by comprehensive evaluation of seven individual oral functions with complex links to performance of oral cavity activities. However, as mentioned previously, many previous reports have linked a single oral function, such as occlusal status, masticatory function, or remaining teeth, to nutritional status [23,24,25,26,35,36]. In terms of nutritional assessment, studies are investigating the relationship between certain oral functions and nutritional status using a simple nutritional assessment such as the Mini Nutritional Assessment^®^-Short Form or the dietary variety score [27,28], but few studies have evaluated the relationship between oral function and intake of nutrients. This study differs from previous studies in that the BDHQ was used for the nutritional assessment, which calculates daily nutrient intake from dietary questionnaire responses. To the best of our knowledge, this study is the first report on the association between oral hypofunction by comprehensive evaluation and nutritional status focusing on protein intake in local community-dwelling adults over 40 years of age. Daily protein intake was not significantly lower in the oral hypofunction group than in the no oral hypofunction group, but serum albumin was significantly lower, showing a similar trend to previous reports [28]. Considering the half-life of serum albumin, this difference may be attributed to the amount of protein stored in the body through daily dietary consumption.

In Japan, the oral hypofunction evaluation approach proposed by the Japanese Society of Gerodontology is still in development. Regarding the evaluation, there are criticisms about the validity of the current seven-item evaluation and the standard value of the examinations. Reviewing the evaluation approach has been considered necessary from the beginning and is still being discussed. Oral hypofunction is diagnosed, when three or more of these seven items measurements are poorer than the normal values, as defined by the Japanese Society of Gerodontology [13]. In order to simplify the evaluation, reducing the number of evaluation items for oral hypofunction has been suggested. In the present study, no examination for oral hypofunction showed a statistically significant relationship with low protein intake; however, the diagnosis of oral hypofunction and number of items used for oral hypofunction evaluation were statistically associated with low protein intake. This suggests that when protein intake is used as an outcome, no evaluations should be actively excluded, and it may be better to evaluate oral health and function comprehensively. The number of indicators of poor functioning in the oral hypofunction evaluation in the under-DG group was 2.8 ± 1.4 (mean ± standard deviation) in this study. Thus, three or more out of seven evaluation items may be appropriate as a cutoff from the perspective of protein intake. The standard value of the examinations for oral hypofunction proposed were defined and primarily based on past reports obtained from studies in young people [13], and there is criticism that these evaluation standards are high. However, we were able to isolate participants with oral hypofunction and those with low protein intake by using these evaluation standards in the present study of participants over 40 years of age. Thus, the current cutoff value does not seem to be unreasonable. Future studies should clarify the validity of the number of examination items for oral hypofunction and the optimal cutoff value.

This study has several limitations. This study was a cross-sectional study with data from 2019 only. Thus, a long-term longitudinal study is essential to determine the extent to which maintaining oral function contributes to nutritional status. The local condition of oral tissues with chronic inflammation may be related to oral function, and local inflammation may deplete protein. However, these relationships were not investigated in this study. It is worthwhile to study the percentage of total energy intake of the three major macronutrients and total protein intake, as well as the intake of specific food groups high in protein in participants with oral hypofunction. Thus, a detailed study on oral hypofunction and the intake of foods composed mainly of protein is necessary in the future. This analysis of oral hypofunction and the intake of specific foods may allow healthcare professions to develop nutritional guidance in dental clinics, since dental clinics have been reported to be suitable places for providing nutritional guidance, as well as restoring and maintaining oral function [38].

## 5. Conclusions

This study was the first to examine nutrition by considering oral function as a whole. Oral hypofunction diagnosed by comprehensive evaluation was associated with targeted protein intake for sarcopenia and frailty prevention in middle-aged and older community-dwelling adults. Healthcare professionals working in dental clinics may be able to apply this knowledge while evaluating patients, though a longitudinal study is needed to further elucidate oral function for the prevention of sarcopenia and frailty.

## Figures and Tables

**Table 1:** The seven-item evaluation for diagnosis of oral hypofunction in this study.

	Evaluation	Criteria
1	Poor oral hygiene	TCI was more than 50%.
2	Oral dryness	The oral moisture checker value was less than 27.
3	Occlusal force	The number of remaining teeth was less than 20.
4	ODK (/pa/, /ta/, /ka/)	The rate of repetitions performed was less than 6 per second.
5	Tongue pressure	Tongue pressure was less than 30 kPa.
6	Masticatory function	The participants answered difficulty chewing the hard foods.
7	Swallowing function	The total score of EAT-10 was more than 3.

Abbreviations: TCI, Tongue Coating Index; ODK, oral diadochokinesis; EAT-10, 10-item Eating Assessment Tool.

**Table 2:** Physical characteristics, examination results for oral hypofunction, and social background in groups with and without oral hypofunction by sex.

	Men (*n* = 382)			Women (*n* = 622)		
	No Oral Hypofunction (*n* = 202)	Oral Hypofunction (*n* = 180)	*p-*Value	No Oral Hypofunction (*n* = 333)	Oral Hypofunction (*n* = 289)	*p-*Value
Age (y)	63.3 ± 11.3	72.3 ± 9.2	<0.0001 ^††^	64.2 ± 10.0	74.0 ± 9.3	<0.0001
BW (kg)	65.6 ± 10.0	63.4 ± 8.8	0.0218	54.7 ± 9.6	51.7 ± 8.6	<0.0001
BMI (kg/m^2^)	23.7 ± 3.1	23.5 ± 2.8	0.5786	23.1 ± 3.7	23.0 ± 3.5	0.7556
SMI (kg/m^2^)	7.6 ± 0.7	7.3 ± 0.7	<0.0001	6.1 ± 0.7	5.8 ± 0.6	<0.0001
Oral hygiene (%)	44.8 ± 29.4	63.3 ± 29.3	<0.0001	33.6 ± 26.9	52.0 ± 31.6	<0.0001
Oral dryness	27.6 ± 2.4	26.6 ± 2.7	<0.0001	27.2 ± 2.3	26.3 ± 2.5	<0.0001
Occlusal force (number)	24.5 ± 6.2	14.3 ± 9.7	<0.0001	24.0 ± 6.2	13.9 ± 9.8	<0.0001
ODK (pa) (/s)	6.3 ± 1.1	5.7 ± 1.1	<0.0001	6.5 ± 0.7	5.7 ± 0.8	<0.0001
ODK (ta) (/s)	6.4 ± 0.9	5.7 ± 1.0	<0.0001	6.6 ± 0.8	5.7 ± 0.8	<0.0001
ODK (ka) (/s)	5.8 ± 1.1	5.0 ± 1.1	<0.0001	6.2 ± 0.7	5.3 ± 0.8	<0.0001
Tongue pressure (kPa)	40 ± 8.9	32.2 ± 10.8	<0.0001	37.6 ± 7.4	30.4 ± 10.0	<0.0001
Masticatory function (*n*)	3	49	<0.0001 ^†^	24	93	<0.0001
Swallowing function	0.6 ± 1.2	1.6 ± 2.8	<0.0001	0.8 ± 2.0	1.7 ± 3.0	<0.0001
Number of indicators of poor functioning in the oral hypofunction evaluation (*n*)	1.5 ± 0.7	3.8 ± 0.9	<0.0001	1.3 ± 0.7	3.8 ± 0.9	<0.0001
Educational history (y)	13.5 ± 2.6	12.0 ± 2.7	<0.0001	12.4 ± 2.0	11.0 ± 1.9	<0.0001
Living alone (*n*)	25	37	0.0305 ^†^	64	98	<0.0001 ^†^

^††^: Welch’s test, ^†^: Chi-square test. Abbreviations: BW, body weight; BMI, body mass index; SMI, skeletal muscle mass index; ODK, oral diadochokinesis; *p*-values were calculated using student’s *t*-test, Welch’s test, and the Chi-square test. Continuous variables are ex pressed as mean ± standard deviation. Nominal variables are indicated by *n* values.

**Table 3:** Food intake characteristics and albumin serological status in groups with and without oral hypofunction.

	Men (*n* = 382)			Women (*n* = 622)		
	No Oral Hypofunction (*n* = 202)	Oral Hypofunction (*n* = 180)	*p-*Value	No Oral Hypofunction (*n* = 333)	Oral Hypofunction (*n* = 289)	*p-*Value
Energy (kcal)	2099.7 ± 554.7	2105.8 ± 584.6	0.9165	1672.8 ± 454.6	1770.2 ± 524.3	0.0143 ^††^
Protein (% energy)	15.4 ± 2.9	15.2 ± 2.9	0.4966	17.0 ± 3.0	17.0 ± 3.2	0.9704
Fat (% energy)	26.7 ± 5.5	25.5 ± 5.3	0.0267	29.7 ± 5.3	28.8 ± 5.1	0.0358
Carbohydrate (% energy)	49.4 ± 8.7	50.7 ± 8.6	0.1453	51.2 ± 7.5	52.4 ± 7.4	0.0333
Total protein (g/day)	81.4 ± 16.5	81.2 ± 15.6	0.8920	73.7 ± 13.0	74.1 ± 14.6	0.6872
Cereals (g/day)	440.2 ± 133.8	426.7 ± 117.2	0.2970	320.2 ± 96.0	336.0 ± 98.9	0.0451
Potatoes (g/day)	50.2 ± 44.0	58.7 ± 56.0	0.1150	59.0 ± 41.5	61.2 ± 50.2	0.5417
Sugar (g/day)	4.6 ± 3.7	5.5 ± 4.4	0.0303	4.1 ± 2.9	4.2 ± 2.8	0.8778
Beans (g/day)	90.4 ± 47.6	91.3 ± 57.0	0.8671 ^††^	89.4 ± 48.2	80.6 ± 45.1	0.0197
Vegetables (green and yellow) (g/day)	120.0 ± 78.1	129.6 ± 78.0	0.2272	133.8 ± 70.0	136.2 ± 68.1	0.6677
Vegetables (other) (g/day)	191.3 ± 107.0	188.0 ± 94.1	0.7472	197.6 ± 88.9	179.2 ± 83.8	0.0083
Fruits (g/day)	111.3 ± 102.2	118.9 ± 90.1	0.4492	134.6 ± 90.2	150.5 ± 103.9	0.0415
Seafoods (g/day)	91.0 ± 50.3	95.9 ± 57.5	0.3732	83.1 ± 41.3	87.8 ± 53.2	0.2085 ^††^
Meats (g/day)	85.8 ± 38.6	76.9 ± 38.8	0.0265	75.7 ± 33.0	72.5 ± 41.8	0.2852
Eggs (g/day)	54.9 ± 27.5	54.5 ± 30.3	0.8941	46.8 ± 23.6	47.0 ± 23.0	0.9371
Dairy products (g/day)	149.3 ± 110.4	154.0 ± 118.1	0.6857	151.9 ± 87.2	158.2 ± 97.4	0.3967
Oil and fat (g/day)	13.4 ± 5.3	12.4 ± 5.4	0.0810	11.6 ± 4.9	10.8 ± 5.0	0.0466
Sweets (g/day)	36.6 ± 27.8	43.6 ± 37.7	0.0403 ^††^	45.6 ± 30.2	41.5 ± 35.8	0.1193
Beverages (g/day)	877.4 ± 375.9	834.4 ± 359.5	0.2559	673.8 ± 298.2	664.1 ± 261.0	0.6685
Seasoning and spices (g/day)	285.2 ± 126.5	315.6 ± 122.1	0.0178	222.7 ± 5.6	236.3 ± 6.0	0.0945
Albumin (g/dL)	4.373 ± 0.3	4.243 ± 0.3	<0.0001	4.348 ± 0.3	4.259 ± 0.3	<0.0001

^††^: Welch’s test; *p*-values were calculated using student’s *t*-test and, Welch’s test. Continuous variables are expressed as mean ± standard deviation.

**Table 4:** Physical characteristics and social background according to over-DG and under-DG protein intake.

	Men (*n* = 382)			Women (*n* = 622)		
	Over DG (*n* = 240)	Under DG (*n* = 142)	*p-*Value	Over DG (*n* = 495)	Under DG (*n* = 127)	*p*-Value
Age (y)	67.3 ± 11.8	68.0 ± 10.5	0.5744	68.6 ± 10.8	69.4 ± 11.1	0.4326
BW (kg)	64.4 ± 9.4	65.0 ± 9.6	0.5515	53.2 ± 9.2	53.6 ± 9.6	0.7072
BMI (kg/m^2^)	23.6 ± 2.9	23.6 ± 3.1	0.8419	23.0 ± 3.5	23.4 ± 4.1	0.2963
SMI (kg/m^2^)	7.5 ± 0.7	7.5 ± 0.7	0.6731	6.0 ± 0.7	6.0 ± 0.7	0.7887
Energy (kcal)	2151.4 ± 548.2	2019.9 ± 593.5	0.0286	1719.1 ± 484.7	1713.9 ± 513.4	0.9148
Protein (% energy)	15.4 ± 2.9	15.2 ± 3.0	<0.0001	18.0 ± 2.6	13.1 ± 1.5	<0.0001
Fat (% energy)	28.3 ± 4.7	22.4 ± 4.7	<0.0001	30.3 ± 4.7	25.3 ± 5.4	<0.0001
Carbohydrate (% energy)	48.5 ± 7.3	52.7 ± 10.1	<0.0001	50.1 ± 6.3	58.5 ± 7.7	<0.0001
Total protein (g/day)	89.5 ± 13.3	67.5 ± 9.5	<0.0001	78.1 ± 11.6	57.5 ± 7.4	<0.0001
Albumin (g/dL)	4.3 ± 0.3	4.3 ± 0.3	0.5114	4.3 ± 0.3	4.3 ± 0.3	0.6465
Educational history (y)	13.1 ± 2.8	12.4 ± 2.7	0.0100	11.8 ± 2.1	11.4 ± 2.2	0.0471
Living alone (n)	33	29	0.0909 ^†^	129	33	0.9860 ^†^

^†^: Chi-square test; Abbreviations: BW, body weight; BMI, body mass index; SMI, skeletal muscle mass index; *p*-values were calculated using student’s *t*-test and Chi-square test. Continuous variables are expressed as mean ± standard deviation. The nominal variables are indicated by *n* value.

**Table 5:** Results of examination for oral hypofunction in groups according to over-DG and under-DG protein intake.

	Men (*n* = 382)			Women (*n* = 622)			Total		
	Over DG (*n* = 240)	Under DG (*n* = 142)	*p*-Value	Over DG (*n* = 495)	Under DG (*n*= 127)	*p-*Value	Over DG (*n* = 735)	Under DG (*n* = 269)	*p*-Value
Oral hypofunction (*n*)	97	83	0.0006 ^†^	219	70	0.0284 ^†^	316	153	<0.0001 ^†^
Number of indicators of poor functioning in the oral hypofunction evaluation (*n*)	2.3 ± 1.4	2.9 ± 1.4	0.0007	2.4 ± 1.5	2.8 ± 1.4	0.0054	2.4 ± 1.4	2.8 ± 1.4	<0.0001
Oral hygiene (%)	51.7 ± 30.1	56.6 ± 31.6	0.1347	42.4 ± 30.4	41.3 ± 31.4	0.7295	45.4 ± 30.6	49.4 ± 32.4	0.0756
Oral dryness	27.1 ± 2.5	27.0 ± 2.7	0.7519	26.8 ± 2.4	26.7 ± 2.4	0.8187	26.9 ± 2.5	26.9 ± 2.5	0.9818
Occlusal force (number)	20.4 ± 9.5	18.5 ± 9.6	0.0550	19.8 ± 9.3	17.2 ± 10.1	0.0054	19.8 ± 9.3	17.2 ± 10.1	0.0015
ODK (/pa/) (syllables/sec)	6.0 ± 1.3	6.0 ± 0.9	0.9190	6.2 ± 0.9	6.0 ± 0.9	0.0014	6.1 ± 1.0	6.0 ± 0.9	0.0316
ODK (/ta/) (syllables/sec)	6.1 ± 1.1	6.0 ± 1.0	0.1798	6.2 ± 0.9	6.1 ± 0.9	0.1444	6.2 ± 1.0	6.0 ± 0.9	0.0193
ODK (/ka/) (syllables/sec)	5.5 ± 1.2	5.4 ± 1.1	0.3183	5.8 ± 0.9	5.7 ± 0.8	0.1101	5.7 ± 1.0	5.5 ± 1.0	0.0005
Tongue pressure (kPa)	36.9 ± 10.6	36.0 ± 10.7	0.4586	34.2 ± 9.5	34.4 ± 9.1	0.8632	35.1 ± 10.0	35.2 ± 10.0	0.8098
Masticatory function (*n*)	29	23	0.2572 ^†^	88	29	0.1933 ^†^	117	52	0.2006 ^†^
Swallowing function	1.1 ± 2.1	1.0 ± 2.2	0.9727	1.3 ± 2.3	1.5 ± 3.3	0.1283	1.1 ± 2.2	1.3 ± 2.8	0.3458

^†^: Chi-square test; Abbreviations: ODK, oral diadochokinesis; *p*-values were calculated using student’s *t*-test and the Chi-square test. Continuous variables are expressed as mean ± standard deviation. The nominal variables are indicated by the *n* value.

**Table 6:** Odds ratio for protein intake according to oral hypofunction and evaluations for oral hypofunction.

	Men		Women		Total	
	Crude OR	95% CI	Crude OR	95% CI	Adjusted OR ^a^	95% CI
Oral hypofunction	2.07	1.36–3.16	1.55	1.05–2.29	1.70	1.21–2.35
Number of indicators of poor functioning in the oral hypofunction evaluation	1.30	1.11–1.51	1.20	1.05–1.37	1.24	1.10–1.40
Oral hygiene	1.00	1.00–1.01	1.00	0.99–1.00	1.00	1.00–1.01
Oral dryness	0.99	0.91–1.07	0.99	0.91–1.07	0.99	0.93–1.05
Occlusal force (number)	0.98	0.96–1.01	0.97	0.95–1.00	0.98	0.96–1.00
ODK (/pa/)	1.18	0.87–1.61	0.65	0.47–0.90	0.95	0.75–1.19
ODK (/ta/)	0.80	0.53–1.20	1.25	0.81–1.92	0.94	0.69–1.27
ODK (/ka/)	1.02	0.75–1.39	1.00	0.67–1.50	1.00	0.78–1.30
Tongue pressure	1.00	0.97–1.02	1.01	0.99–1.04	1.00	0.99–1.02
Masticatory function	1.32	0.70–2.52	1.06	0.63–1.77	1.10	0.72–1.65
Swallowing function	0.98	0.98–1.09	1.04	0.97–1.14	1.03	0.96–1.09

Abbreviations: OR, odds ratio; 95% CI, 95% confidence interval; ODK, oral diadochokinesis; **^a^** Values adjusted for age, sex, BMI, energy intake, heart disease, smoking, appetite, living alone, vitality, and education history.

## Data Availability

The data presented in this study are available on request from the corresponding author.

## References

[1] Ministry of Health, Labour and Welfare (2020). The White Paper on Ministry of Health, Labour and Welfare. https://www.mhlw.go.jp/content/000735866.pdf.

[2] Iijima K., Arai H., Akishita M., Endo T., Ogasawara K., Kashihara N., Hayashi Y.K., Yumura W., Yokode M., Ouchi Y. (2021). Toward the development of a vibrant, super-aged society: The future of medicine and society in Japan. Geriatr. Gerontol. Int..

[3] Pinedo-Villanueva R., Westbury L.D., Syddall H.E., Sanchez-Santos M.T., Dennison E.M., Robinson S.M., Cooper C. (2019). Health Care Costs Associated With Muscle Weakness: A UK Population-Based Estimate. Calcif. Tissue Int..

[4] Houston D.K., Nicklas B.J., Ding J., Harris T.B., Tylavsky F.A., Newman A.B., Lee J.S., Sahyoun N.R., Visser M., Kritchevsky S.B. (2008). Dietary protein intake is associated with lean mass change in older, community-dwelling adults: The Health, Aging, and Body Composition (Health ABC) Study. Am. J. Clin. Nutr..

[5] Nanri H., Yamada Y., Yoshida T., Okabe Y., Nozawa Y., Itoi A., Yoshimura E., Watanabe Y., Yamaguchi M., Yokoyama K. (2018). Sex Difference in the Association Between Protein Intake and Frailty: Assessed Using the Kihon Checklist Indexes Among Older Adults. J. Am. Med. Dir. Assoc..

[6] Feart C. (2019). Nutrition and frailty: Current knowledge. Prog. Neuro-Psychopharmacol. Biol. Psychiatry.

[7] Kobayashi S., Asakura K., Suga H., Sasaki S., Three-Generation Study of Women on Diets and Health Study Group (2013). High protein intake is associated with low prevalence of frailty among old Japanese women: A multicenter cross-sectional study. Nutr. J..

[8] Kobayashi S., Suga H., Sasaki S. (2017). Diet with a combination of high protein and high total antioxidant capacity is strongly associated with low prevalence of frailty among old Japanese women: A multicenter cross-sectional study. Nutr. J..

[9] Coelho-Júnior H.J., Rodrigues B., Uchida M., Marzetti E. (2018). Low Protein Intake Is Associated with Frailty in Older Adults: A Systematic Review and Meta-Analysis of Observational Studies. Nutrients.

[10] Feng Z., Lugtenberg M., Franse C., Fang X., Hu S., Jin C., Raat H. (2017). Risk factors and protective factors associated with incident or increase of frailty among community-dwelling older adults: A systematic review of longitudinal studies. PLoS ONE.

[11] Ministry of Health, Labour and Welfare (2020). Dietary Reference Intakes for Japanese (2020). https://www.mhlw.go.jp/content/10904750/000586553.pdf.

[12] Ministry of Health, Labour and Welfare (2020). Eat Well Preveny Frailty. https://www.mhlw.go.jp/content/000620855.pdf.

[13] Minakuchi S., Tsuga K., Ikebe K., Ueda T., Tamura F., Nagao K., Furuya J., Matsuo K., Yamamoto K., Kanazawa M. (2018). Oral hypofunction in the older population: Position paper of the Japanese Society of Gerodontology in 2016. Gerodontology.

[14] Hakeem F.F., Bernabé E., Sabbah W. (2019). Association between oral health and frailty: A systematic review of longitudinal studies. Gerodontology.

[15] Horibe Y., Ueda T., Watanabe Y., Motokawa K., Edahiro A., Hirano H., Shirobe M., Ogami K., Kawai H., Obuchi S. (2018). A 2-year longitudinal study of the relationship between masticatory function and progression to frailty or pre-frailty among community-dwelling Japanese aged 65 and older. J. Oral Rehabil..

[16] Murakami M., Hirano H., Watanabe Y., Sakai K., Kim H., Katakura A. (2015). Relationship between chewing ability and sarcopenia in Japanese community-dwelling older adults. Geriatr. Gerontol. Int..

[17] Satake A., Kobayashi W., Tamura Y., Oyama T., Fukuta H., Inui A., Sawada K., Ihara K., Noguchi T., Murashita K. (2019). Effects of oral environment on frailty: Particular relevance of tongue pressure. Clin. Interv. Aging.

[18] Machida N., Tohara H., Hara K., Kumakura A., Wakasugi Y., Nakane A., Minakuchi S. (2017). Effects of aging and sarcopenia on tongue pressure and jaw-opening force. Geriatr. Gerontol. Int..

[19] Hoeksema A.R., Spoorenberg S., Peters L.L., Meijer H., Raghoebar G.M., Vissink A., Wynia K., Visser A. (2017). Elderly with remaining teeth report less frailty and better quality of life than edentulous elderly: A cross-sectional study. Oral Dis..

[20] Shimazaki Y., Nonoyama T., Tsushita K., Arai H., Matsushita K., Uchibori N. (2020). Oral hypofunction and its association with frailty in community-dwelling older people. Geriatr. Gerontol. Int..

[21] Kugimiya Y., Iwasaki M., Ohara Y., Motokawa K., Edahiro A., Shirobe M., Watanabe Y., Obuchi S., Kawai H., Fujiwara Y. (2021). Relationship between Oral Hypofunction and Sarcopenia in Community-Dwelling Older Adults: The Otassha Study. Int. J. Environ. Res. Public Health.

[22] Nakamura M., Hamada T., Tanaka A., Nishi K., Kume K., Goto Y., Beppu M., Hijioka H., Higashi Y., Tabata H. (2021). Association of Oral Hypofunction with Frailty, Sarcopenia, and Mild Cognitive Impairment: A Cross-Sectional Study of Community-Dwelling Japanese Older Adults. J. Clin. Med..

[23] Motokawa K., Mikami Y., Shirobe M., Edahiro A., Ohara Y., Iwasaki M., Watanabe Y., Kawai H., Kera T., Obuchi S. (2021). Relationship between Chewing Ability and Nutritional Status in Japanese Older Adults: A Cross-Sectional Study. Int. J. Environ. Res. Public Health.

[24] Okamoto N., Amano N., Nakamura T., Yanagi M. (2019). Relationship between tooth loss, low masticatory ability, and nutritional indices in the elderly: A cross-sectional study. BMC Oral Health.

[25] Nomura Y., Kakuta E., Okada A., Otsuka R., Shimada M., Tomizawa Y., Taguchi C., Arikawa K., Daikoku H., Sato T. (2020). Effects of self-assessed chewing ability, tooth loss and serum albumin on mortality in 80-year-old individuals: A 20-year follow-up study. BMC Oral Health.

[26] Hollis J.H. (2018). The effect of mastication on food intake, satiety and body weight. Physiol. Behav..

[27] Hoshino D., Hirano H., Edahiro A., Motokawa K., Shirobe M., Watanabe Y., Motohashi Y., Ohara Y., Iwasaki M., Maruoka Y. (2021). Association between Oral Frailty and Dietary Variety among Community-Dwelling Older Persons: A Cross-Sectional Study. J. Nutr. Health Aging.

[28] Iwasaki M., Motokawa K., Watanabe Y., Shirobe M., Inagaki H., Edahiro A., Ohara Y., Hirano H., Shinkai S., Awata S. (2020). Association between Oral Frailty and Nutritional Status among Community-Dwelling Older Adults: The Takashimadaira Study. J. Nutr. Health Aging.

[29] Iwasaki M., Motokawa K., Watanabe Y., Shirobe M., Ohara Y., Edahiro A., Kawai H., Fujiwara Y., Kim H., Ihara K. (2021). Oral hypofunction and malnutrition among community-dwelling older adults: Evidence from the Otassha study. Gerodontology.

[30] Shimizu T., Ueda T., Sakurai K. (2007). New method for evaluation of tongue-coating status. J. Oral Rehabil.

[31] Tanaka T., Takahashi K., Hirano H., Kikutani T., Watanabe Y., Ohara Y., Furuya H., Tetsuo T., Akishita M., Iijima K. (2018). Oral Frailty as a Risk Factor for Physical Frailty and Mortality in Community-Dwelling Elderly. J. Gerontol. Biol. Sci. Med. Sci..

[32] Kobayashi S., Murakami K., Sasaki S., Okubo H., Hirota N., Notsu A., Fukui M., Date C. (2011). Comparison of relative validity of food group intakes estimated by comprehensive and brief-type self-administered diet history questionnaires against 16 d dietary records in Japanese adults. Public Health Nutr..

[33] Kaimoto K., Yamashita M., Suzuki T., Makizako H., Koriyama C., Kubozono T., Takenaka T., Ohishi M., Kanouchi H., The Tarumizu Study Diet Group (2021). Association of Protein and Magnesium Intake with Prevalence of Prefrailty and Frailty in Community-Dwelling Older Japanese Women. J. Nutr. Sci. Vitaminol..

[34] Coelho-Júnior H.J., Calvani R., Picca A., Gonçalves I.O., Landi F., Bernabei R., Cesari M., Uchida M.C., Marzetti E. (2020). Protein-Related Dietary Parameters and Frailty Status in Older Community-Dwellers across Different Frailty Instruments. Nutrients.

[35] Yoshida M., Kikutani T., Yoshikawa M., Tsuga K., Kimura M., Akagawa Y. (2011). Correlation between dental and nutritional status in community-dwelling elderly Japanese. Geriatr. Gerontol. Int..

[36] Yoshihara A., Watanabe R., Nishimuta M., Hanada N., Miyazaki H. (2005). The relationship between dietary intake and the number of teeth in elderly Japanese subjects. Gerodontology.

[37] Takeuchi H., Terada M., Kobayashi K., Uraguchi M., Nomura Y., Hanada N. (2019). Influences of Masticatory Function Recovery Combined with Health Guidance on Body Composition and Metabolic Parameters. Open Dent. J..

[38] Nomura Y., Takeuchi H., Shigemoto S., Okada A., Shigeta Y., Ogawa T., Hanada N. (2017). Secondary Endpoint of the Prosthodontics. Int. J. Clin. Case Stud..

[39] Nomura Y., Ishii Y., Suzuki S., Morita K., Suzuki A., Suzuki S., Tanabe J., Ishiwata Y., Yamakawa K., Chiba Y. (2020). Nutritional Status and Oral Frailty: A Community Based Study. Nutrients.

